# Association between Alcohol Consumption and Cancers in the Chinese Population—A Systematic Review and Meta-Analysis

**DOI:** 10.1371/journal.pone.0018776

**Published:** 2011-04-15

**Authors:** Ying Li, Huan Yang, Jia Cao

**Affiliations:** 1 Department of Social Medicine and Health Service Management, Third Military Medical University, Chongqing, China; 2 Department of Hygienic Toxicology, Key Lab of Medical Protection for Electromagnetic Radiation, Ministry of Education of China, Third Military Medical University, Chongqing, China; Alberta Research Centre for Health Evidence, University of Alberta, Canada

## Abstract

**Background:**

Alcohol consumption is increasing worldwide and is associated with numerous cancers. This systematic review examined the role of alcohol in the incidence of cancer in the Chinese population.

**Methods:**

Medline/PubMed, EMBASE, CNKI and VIP were searched to identify relevant studies. Cohort and case-control studies on the effect of alcohol use on cancers in Chinese were included. Study quality was evaluated using the Newcastle-Ottawa Scale. Data were independently abstracted by two reviewers. Odds ratios (OR) or relative risks (RR) were pooled using RevMan 5.0. Heterogeneity was evaluated using the Q test and I-squared statistic. P<.01 was considered statistically significant.

**Results:**

Pooled results from cohort studies indicated that alcohol consumption was not associated with gastric cancer, esophageal cancers (EC) or lung cancer. Meta-analysis of case-control studies showed that alcohol consumption was a significant risk factor for five cancers; the pooled ORs were 1.79 (99% CI, 1.47–2.17) EC, 1.40 (99% CI, 1.19–1.64) gastric cancer, 1.56 (99% CI, 1.16–2.09) hepatocellular carcinoma, 1.21 (99% CI, 1.00–1.46) nasopharyngeal cancer and 1.71 (99% CI, 1.20–2.44) oral cancer. Pooled ORs of the case-control studies showed that alcohol consumption was protective for female breast cancer and gallbladder cancer: OR 0.76 (99% CI, 0.60–0.97) and 0.70 (99% CI, 0.49–1.00) respectively. There was no significant correlation between alcohol consumption and lung cancer, colorectal cancer, pancreatic cancer, cancer of the ampulla of Vater, prostate cancer or extrahepatic cholangiocarcinoma. Combined results of case-control and cohort studies showed that alcohol consumption was associated with 1.78- and 1.40-fold higher risks of EC and gastric cancer but was not significantly associated with lung cancer.

**Conclusions:**

Health programs focused on limiting alcohol intake may be important for cancer control in China. Further studies are needed to examine the interaction between alcohol consumption and other risk factors for cancers in Chinese and other populations.

## Introduction

Alcohol has a long history of use and abuse in numerous cultures around the world, and alcohol consumption has been increasing rapidly in many countries [1]. In particular, there has been a rapid increase in the consumption of alcohol in China. According to the data from national surveys conducted in 1999, 2002 and 2007, alcohol consumption increased 12.8% for males (from 35.09% to 39.6%) and 73.1% for females (from 2.58% to 4.5%) between 1991 and 2002; alcohol consumption doubled for males (from 39.6% to 84.1%) and increased 6.5 times (from 4.5% to 29.3% for females) for females during the five years between 2002 and 2007 [2]–[3]. It is estimated that there are currently more than 500 million Chinese who regularly consume alcohol [3]. The WHO pointed out that the “safe limit” of ethanol consumption is less than 40 g for males and 20 g for females per day [4]. The current suggested alcohol limit in China is less than 25 g for males and 15 g for females [5]. However, the average Chinese subject who consumes alcohol drinks 41.04 g, which exceeds the international and national limits [3]. The overall heavy drinking rate of adults in China also increased rapidly, from 4.7% in 2002 to 37.04% in 2007, and 65.39% of drinkers have poor health [2]–[3]. With the purpose of addressing the social and medical challenges highlighted by these reports, in 2007, the Chinese government advocated ***limited drinking, scientific drinking and protection of personal health*** [2], but heavy drinking is still common. Moreover, it is clear that alcohol consumption has already become one of the most important dietary habits linked with poor health in contemporary Chinese society.

Ethanol itself is not carcinogenic. However, its first metabolite (acetaldehyde) has recently been shown to be a local carcinogen in humans [6]. Moreover, alcohol consumption is an important risk factor for numerous cancers worldwide [7]. Due to hereditary differences/genetic polymorphisms [8]–[9], the incidence and causes of cancers and the responses to anti-cancer drugs vary substantially among different ethnic groups. Numerous researchers have studied the association between alcohol consumption and cancers in Chinese people [10]–[17]. The potential association between alcohol consumption and cancer risk for the Chinese population is currently being debated in the literature, and a clear consensus of opinion has not emerged.

Although there have been a few meta-analyses based on studies that investigated the association between alcohol and EC, gastric cancer, HCC or lung cancer in Chinese patients [18]–[21], few of these earlier studies systematically analyzed the association between alcohol and some common cancers in the Chinese population. With the aim of understanding the effect of alcohol consumption on the risk of developing various cancers in the Chinese population, we qualitatively and quantitatively reviewed all of the available literature published in English and Chinese regarding the association between alcohol and cancers in Chinese people. We conclude with a series of recommendations regarding future intervention programs and studies.

## Materials and Methods

### Search strategy

Electronic searches of databases and hand searches of other resources were conducted to identify published articles for review. We searched articles (published up to February 2010) from four main databases: Medline/PubMed, EMBASE, CNKI (China National Knowledge Infrastructure) and the VIP database (Chinese Journal of Science and Technology of VIP). These searches included a mixture of free text and index terms to maximize the retrieval of potentially relevant articles. In addition to reviewing the references cited in the retrieved articles, the bibliographies of retrieved papers were searched by hand.

### Selection of studies

Cohort and case-control studies of Chinese subjects were included in the study. The risk factor examined in all of these studies was alcohol consumption. Numerous cancer types were included. The types of alcohol consumed included beer, yellow rice wine, red wine, and hard liquor. We also included studies that were designed with the purpose of analyzing risk factors for cancer in Chinese people other than alcohol but that included alcohol as one factor for which the data were collected and analyzed as a secondary aim. Studies that only provided odds ratios (OR) or relative risk (RR) values and did not provide sufficient original data to calculate the OR or RR; studies that used data reported by earlier studies; systematic reviews; and meta-analyses were excluded from the present meta-analysis.

### Validity assessment

The quality of the primary studies was evaluated using the Newcastle-Ottawa Scale (NOS) [22]. This scale judges the study on three broad perspectives: the selection of the study groups; the comparability of the groups; and the ascertainment of either the exposure or outcome of interest for case-control or cohort studies, respectively.

### Data abstraction

All of the identified studies about alcohol consumption and cancer in Chinese subjects were examined in detail. The data from potentially relevant articles were independently abstracted by two reviewers. Differences were resolved by consensus. For case-control studies, information about the size of the case and control groups, including the number of cases and controls exposed and not exposed to alcohol, were abstracted from each study. For cohort studies, the number of subjects in the cohort and the number of incident cases of cancer in the alcohol-exposed and non-exposed groups were abstracted from each study. When the results of a study were published more than once, only the most complete data were included in the analysis.

### Assessment of heterogeneity and data synthesis

The definitions of drinker and non-drinker were complex and varied widely between studies. In this review, we took participants described as drinking the smallest amount and those who said that they never drink as “non-drinkers”, while the rest of the subjects were classified into the “drinker” category. A qualitative meta-analysis was conducted by summarizing, comparing and contrasting the abstracted data. We calculated the pooled ORs for case-control studies and the RRs for cohort studies separately using RevMan 5.0 software. Heterogeneity was evaluated using the Q test [23] and the I-squared statistic [24]. If the level of heterogeneity was acceptable (p>0.10, or p≤0.10 but I^2^≤50%), the meta-analysis was conducted using a fixed effects model. If significant heterogeneity was found (p≤0.10, I^2^>50%), a random effects model was used for the meta-analysis. Subgroup analyses were also performed to explore the possible reasons for the heterogeneity. Additionally, sensitivity analyses were undertaken to evaluate the stability of the relationship between alcohol consumption and cancer.

For the studies with dose-response analysis, we considered to pool the effect of dose-response. We took the amount of alcohol consumed per week, the duration of drinking or the age at the start drinking as explanatory variables. Because for many studies exposure was reported as a categorical data with a range, we assigned the mid-point of the range as the average length of exposure. For the highest consumption category, we assigned a value equal to half of the width of the previous interval above the uppermost cut-off point. We analyzed dose-response relationship using the method described by Greenland and Longnecker [25]. P<0.01 was chosen as the level of statistical significance for all tests.

## Results

### Description of studies

**Figure 1:**
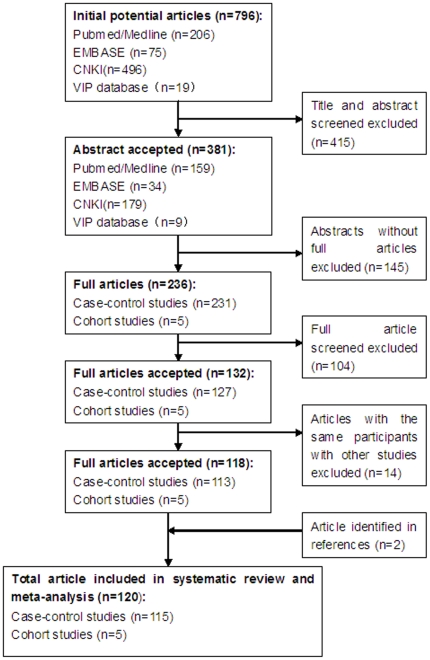
Results of literature search. This figure describes the whole process of searching for articles for inclusion in this systematic review and meta-analysis.

A detailed diagram of the review process is presented in Figure 1. We identified a total of 796 potentially relevant articles. After reviewing the titles, abstracts and full text of the literature, 134 articles were indentified with analysis on the association between alcohol consumption and cancers in Chinese. However, 14 articles were excluded because they involved the same study subjects as other included articles. Finally, 120 studies, which included 181,300 study subjects (including 34,742 cancer patients) from 28 provinces, municipalities and regions, were included in this review. Among these studies, 4 were cohort studies, and 115 were case-control studies [10]–[17], [26]–[135].

The NOS results showed that the median overall score was 7 (range 5 to 9), which indicated that the methodological quality was generally good. We defined studies that scored a 7 or above as having high methodological quality, and we judged that 10 out of the 120 studies to be of low quality (nine studies [49], [56], [66], [71], [82], [97], [112], [132], [135] and one study [46] scored 6 and 5, respectively) primarily due to either no description of the case selection, no definition of controls, a lack of adjusted analysis, or no description of the method of ascertainment for cases and controls.

### Qualitative analysis

In total, the association between alcohol consumption and the incidence of 13 cancers was investigated (see Table 1). Four cancers, including EC, gastric cancer, lung cancer and colorectal cancer, were studied in both case-control studies and cohort studies, while the other cancers were investigated only in case-control studies. The association between alcohol and five cancers, EC, gastric cancer, HCC, pancreatic cancer and lung cancer, were analyzed separately in male patients and female patients in individual studies. In addition to prostate cancer, the effect of alcohol on five other cancers (EC, gastric cancer, HCC, colorectal cancer, and lung cancer) was only investigated in males in some studies. Participants in one study on HCC and all participants in studies on breast cancer were female.

**Table 1:** Studies on the association between alcohol and cancers in Chinese people.

Cancers	No. of studies and study design	Sample size (cases/control)	Participants
Esophageal cancers (EC) [10]–[11], [27]–[59]	34 case-control studies2 cohort studies	10189/60318	Male, FemaleMale and female
Gastric cancer [12]–[13], [38], [49]–[50], [55], [60]–[83]	29 case-control studies2 cohort studies	8645/41580	Male, FemaleMale and female
Hepatocellular carcinoma (HCC) [14], [49], [55], [84]–[98]	18 case-control studies	3812/10927	Male, FemaleMale and female
Colorectal cancer [15], [99]–[108]	10 case-control studies1 cohort studies	4311/6051	Male, FemaleMale and female
Lung cancer [16], [109]–[113]	4 case-control studies2 cohort studies	1104/14731	Male, FemaleMale and female
Breast cancer [17], [114]–[116]	4 case-control studies	1655/2175	Female
Pancreatic cancer [117]–[121]	5 case-control studies	1612/3997	Male, FemaleMale and female
Prostate cancer (PCa) [122]–[125]	4 case-control studies	569/967	Male
Nasopharyngeal cancer(NPC) [126]–[129]	4 case-control studies	1698/1874	Male and female
Oral cancer [130]–[132]	3 case-control studies	347/539	Male, FemaleMale and female
Gallbladder cancer [133]–[134]	2 case-control studies	467/1315	Male and female
Ampulla of Vater cancer [133]–[134]	2 case-control studies	105/1331	Male and female
Extrahepatic cholangiocarcinoma (ECC) [134]–[135]	2 case-control studies	228/753	Male and female

Synergistic effects of alcohol and smoking were analyzed for four cancers in individual studies. Synergistic effects of alcohol and smoking were found for HCC [94]. In addition, a synergistic effect was found for EC in three studies [30], [53]–[54], but another study reported no significant effect of the combination on EC [28]. No synergistic effects were found for gastric cancer [64], [74] or pancreatic cancer [123].

### Quantitative analysis

Heterogeneity tests indicated that studies analyzing the association between nine cancers (EC, gastric cancer, lung cancer, HCC, colorectal cancer, pancreatic cancer, female breast cancer, nasopharyngeal cancer, and cancer of the ampulla of Vater) and alcohol consumption had significant heterogeneity (p<0.10 and I^2^>50%). We therefore conducted meta-analyses using the random effects model. The case-control studies on prostate cancer, oral cancer, gallbladder cancer and ECC lacked heterogeneity (p = 0.24, 0.33, 0.27 and 0.81, respectively) (Table 2). The fixed effects model was used for the meta-analysis of these four cancers.

**Table 2:** Results of meta-analysis of the studies on association between alcohol and cancer in Chinese population.

Cancers	No. of cases (drinker/non-drinker)	No. of controls (drinker/non-drinker)	Variance between studies		Pooled OR/RR (99% CI)	Test for overall effect (p)
			Q(*p*)	I^2^ (%)		
EC (*case-control*)	3764/4366	5343/9206	<0.00001	87	1.79 (1.47, 2.17)	<0.00001
EC (*cohort*)	519/1540	14058/31711	<0.00001	96	1.08 (0.94, 1.23)	0.17
EC(overall)	4283/5906	19401/40917	<0.00001	90	1.78 (1.38, 2.30)	<0.00001
Gastric cancer (*case-control*)	2839/4089	3686/7594	<0.00001	71	1.40 (1.19, 1.64)	<0.00001
Gastric cancer (*cohort*)	490/1227	7368/22932	0.008	86	1.14 (0.99, 1.32)	0.02
Gastric cancer (overall)	3329/5316	11054/30526	<0.00001	73	1.40 (1.20, 1.64)	<0.00001
Lung cancer (*case-control*)	370/333	1179/1071	0.003	79	1.59 (0.86, 2.94)	0.14
Lung cancer (*cohort*)	223/178	6325/6156	0.07	69	1.27 (0.85, 1.91)	0.25
Lung cancer (overall)	593/511	7503/7227	0.004	71	1.39 (0.93, 2.07)	0.03
HCC(*case-control*)	2050/1762	3671/7256	<0.00001	83	1.56 (1.16, 2.09)	0. 0001
Colorectal cancer (*case-control*)	1614/2697	1800/4251	<0.00001	95	1.58 (0.90, 2.76)	0.04
Breast cancer (*case-control*)	266/1379	359/1806	0.08	55	0.76 (0.60,0.97)	0.004
Pancreatic cancer (*case-control*)	452/1154	1005/2975	0.04	60	1.15 (0.97, 1.37)	0.04
Ampulla of Vater cancer (*case-control*)	28/77	467/965	0.04	77	0.68 (0.20, 2.37)	0.43
Prostate cancer (*case-control*)	262/307	415/552	0.24	29	1.17 (0.84, 1.62)	0.23
Nasopharyngeal cancer (*case-control*)	571/1127	536/1338	0.08	55	1.21 (1.00, 1.46)	0.009
Oral cancer (*case-control*)	172/170	243/388	0.33	9	1.71 (1.20, 2.44)	0.0001
Gallbladder cancer (*case-control*)	92/375	355/960	0.27	16	0.70 (0.49, 1.00)	0.009
ECC (*case-control*)	80/148	253/500	0.81	0	1.14 (0.75, 1.75)	0.41

**Figure 2:**
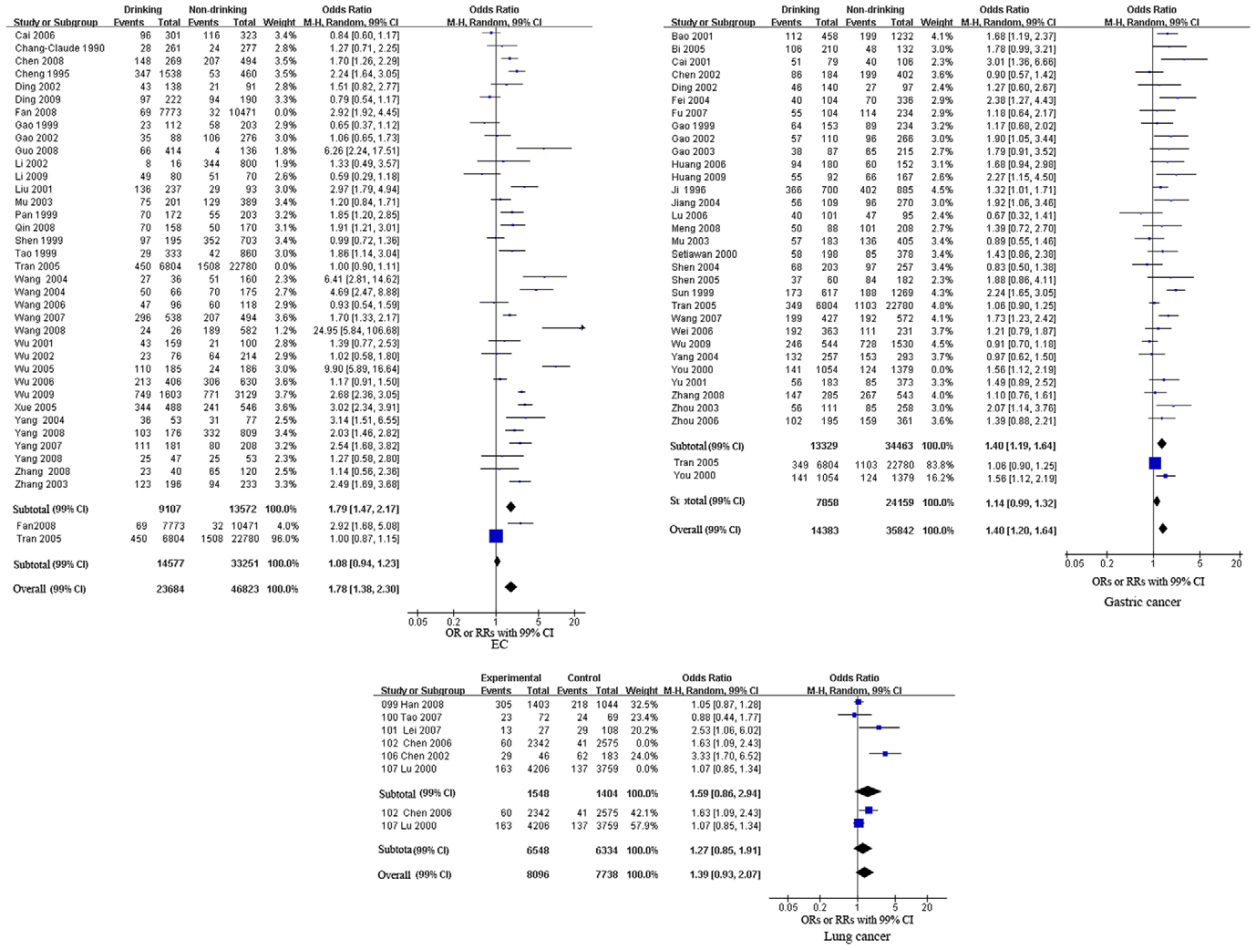
Forest plot of relative risk estimates of incident EC, gastric cancer, and lung cancer by alcohol consumption in Chinese. This figure shows forest plots for the meta-analysis of the association between alcohol consumption and the risk of EC, gastric cancer, and lung cancer. OR and the 99% CI for each cancer was given.

**Figure 3:**
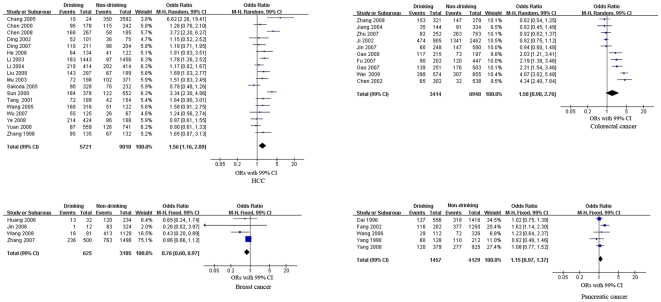
Forest plot of relative risk estimates of incident HCC, colorectal cancer, breast cancer and pancreatic cancer by alcohol consumption in Chinese. This figure shows forest plots for the meta-analysis of the association between alcohol consumption and the risk of HCC, colorectal cancer, breast cancer and pancreatic cancer. OR and the 99% CI for each cancer was given.

**Figure 4:**
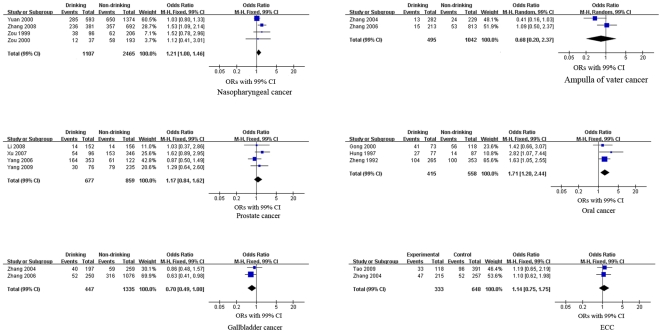
Forest plot of relative risk estimates of incident nasopharyngeal cancer, cancer of the ampulla of Vater, prostate cancer, oral cancer, gallbladder cancer and ECC by alcohol consumption in Chinese. This figure shows the forest plots for the meta-analysis of the association between alcohol consumption and the risk of nasopharyngeal cancer, cancer of the ampulla of Vater, prostate cancer, oral cancer, gallbladder cancer and ECC. OR and the 99% CI for each cancer was given.

We performed meta-analyses of the study design and cancers separately (see Figures 2, 3, 4 and Table 2). The pooled RRs of the cohort studies were 1.08 (99% CI, 0.94–1.23, p = 0.17), 1.14 (99% CI, 0.99–1.32, p = 0.02) and 1.27 (99% CI, 0.85–1.91, p = 0.25) for EC, gastric cancer and lung cancer, respectively. These results indicate that alcohol consumption was not significantly associated with EC, gastric cancer or lung cancer. Meta-analysis of the case-control studies revealed that alcohol consumption was a significant risk factor for five cancers. The pooled ORs were 1.79 (99% CI, 1.47–2.17, p<0.00001) for EC, 1.40 (99% CI, 1.19–1.64, p<0.00001) for gastric cancer, 1.56 (99% CI, 1.16–2.09, p = 0.0001) for HCC, 1.21 (99% CI, 1.00–1.46, p = 0.009) for nasopharyngeal cancer, and 1.71 (99% CI, 1.20–2.44, p<0.0001) for oral cancer. The pooled ORs of the case-control studies showed that alcohol consumption was a protective factor for two cancers, female breast cancer, with an OR of 0.76 (99% CI, 0.60–0.97, p = 0.004) and gallbladder cancer, with an OR of 0.70 (99% CI, 0.49–1.00, p = 0.009) respectively. However, the effects of alcohol consumption on lung cancer, colorectal cancer, pancreatic cancer, cancer of the ampulla of Vater, prostate cancer and ECC were uncertain [pooled OR (99% CI): 1.59 (0.86–2.94), 1.58 (0.90–2.76), 1.15 (0.97–1.37), 0.68 (0.20–2.37), 1.17 (0.84–1.62) and 1.14 (0.75, 1.75), respectively].

We also pooled the results of both case-control and cohort studies for EC, gastric cancer and lung cancer. The overall estimates were 1.78 (99% CI, 1.38–2.30, p<0.00001) for EC, 1.40 (99% CI, 1.20–1.64, p<0.00001) for gastric cancer, and 1.39 (99% CI, 0.93–2.07, p = 0.03) for lung cancer. These results were consistent with pooled ORs for the case-control studies for these three cancers (alcohol consumption was a risk factor for EC and gastric cancer but had no significant association with lung cancer; see Figure 2 and Table 2).

### Subgroup analyses

Given the available data, we conducted subgroup analysis for EC, gastric cancer, HCC and pancreatic cancer after stratifying the participants by sex (male vs. female patients).

The heterogeneity test showed that studies on the effects of alcohol consumption on female EC and gastric cancer and on male pancreatic cancer lacked heterogeneity (p = 0.88, 0.34 and 0.93). Heterogeneity was still found among studies on female HCC and pancreatic cancer and on male EC, HCC and gastric cancer (p = 0.001, 0.10, <0.00001, 0.05 and 0.01, I^2^>50%).

For males, alcohol consumption was a risk factor for EC, HCC and pancreatic cancer but was not significantly associated with gastric cancer and lung cancer. The pooled ORs were 1.82 (99% CI, 1.49–2.22, p<0.00001), 1.56 (99% CI, 1.01–1.62, p = 0.001), 1.71 (99% CI, 1.32–2.20, p<0.00001), 1.12 (99% CI, 0.78–1.59, p = 0.43), and 1.19 (99% CI, 0.91–1.54, p = 0.09), respectively.

However, for females, alcohol consumption was only a risk factor for female gastric cancer and had no significant effect on the other three cancers. The pooled ORs were 1.97 (99% CI, 1.02–4.43, p = 0.005) for gastric cancer, 0.91 (99% CI, 0.47–1.77, p = 0.72) for EC, 1.93 (99% CI, 0.81–4.57, p = 0.05) for HCC, and 1.16 (99% CI, 0.52–2.60, p = 0.63) for pancreatic cancer.

### Sensitivity analysis

We carried out sensitivity analysis for EC, gastric cancer, HCC and lung cancer based on the year of publication and the quality of the study (according to the NOS score).

With regard to EC, gastric cancer and HCC, when six studies [28], [37]–[38], [39], [54], [57] on EC, three studies [38], [69], [83] on gastric cancer and one study [84] on HCC published before 2000 were excluded, the overall strength slightly changed, but the direction of results did not change significantly [the overall estimates were 1.89 (99% CI, 1.41–2.53, p<0.00001) for EC, 1.37 (99% CI, 1.16–1.62, p<0.00001) for gastric cancer, and 1.56 (99% CI, 1.14–2.21, p = 0.0002)for HCC, respectively, when these studies were excluded]. When three studies [46], [49], [56] on EC, three studies [66], [71], [82] on gastric cancer and one study [97] on HCC scoring 6.0 or below on the NOS were excluded, the overall strength slightly changed, but the direction of results did not change significantly [the overall estimates were 1.78 (99% CI, 1.37–2.31, p<0.00001) for EC, 1.40 (99% CI, 1.19–1.65, p<0.00001) for gastric cancer, and 1.55 (99% CI, 1.13–2.13, p = 0.0004) for HCC, respectively, when these studies were excluded]. Similarly, for lung cancer, the strength and direction of results did not changed significantly after excluding one study [112] that scored 6.0. Regarding pancreatic cancer, when two studies [120]–[121] published before 2000 were excluded, the strength increased [pooled OR (99% CI): 1.30 (1.04–1.63)].

### Dose-response analysis

The dose-response relationship between alcohol consumption and four cancers were analyzed in some studies: there are 5 studies on alcohol consumption (g) per week/day/month and EC [11], [39], [49], [54], [57], 6 studies on duration of alcohol consumption (year) and EC [11], [39]–[40], [54], [57], [59], 5 studies on duration of alcohol consumption (year) and gastric cancer [61], [64], [69], [74], [83], 3 studies on initial age at starting alcohol consumption and gastric cancer [64], [69], [74], 4 studies on alcohol consumption (g) per week/day/month and gastric cancer [64], [69], [74], [83], 4 studies on initial age at starting alcohol consumption and HCC [84], [92], [94]–[95], 3 studies on duration of alcohol consumption (year) and HCC [84], [93]–[94], 6 studies on alcohol consumption (g) per week/day/month and HCC [84], [92]–[95], [97], and 2 studies on alcohol consumption (g) per week/day/month and pancreatic cancer [119]–[120]. We pooled study data before trend analysis using the “pool-first” method. But no data indicated a monotonic increasing function relating alcohol consumption with any cancer risk. There is no significant dose-response relationship between alcohol consumption and these cancers in Chinese. We therefore didn't pool the slopes of these studies.

## Discussion

In China, alcohol consumption has an important place in many cultural celebrations, and there has been a drinking culture in China for at least 7000 years. In addition to consuming spirits during festive or happy occasions for celebration, it is also common to drink with business partners or would-be friends in order to solidify the partnership, especially in northern China. Thus, alcohol has played and is playing an increasingly common role in Chinese culture.

All of the previous meta-analyses based on international studies or studies in China consistently found alcohol to be a risk factor for EC (pooled RR = 4.2, pooled ORs = 2.30 and 2.16, respectively) [18], [136]–[137], gastric cancer (pooled RR = 1.32, pooled ORs = 2.03 and 1.90, respectively) [19], [136]–[138] and HCC (RR = 1.86 and OR = 1.872, respectively) [21], [136]. Our meta-analysis provides further support that alcohol consumption is a significant risk factor for EC, gastric cancer, and HCC in the Chinese population. Our subgroup analysis found that the effect of alcohol on the development of gastric cancer and HCC likely depends on sex: alcohol consumption is a risk factor for EC and HCC in male Chinese subjects (pooled ORs = 1.82 and 1.56 respectively), but not for the females; however, it is a risk factor for gastric cancer in Chinese females (pooled OR: 1.97), but not Chinese males. Regarding colorectal cancer, earlier meta-analyses found that alcohol consumption was a risk factor for colorectal cancer (RR = 1.32 and 1.38) worldwide [136], [139]. The present meta-analysis similarly found that alcohol is not a risk factor for colorectal cancer in the Chinese population (p = 0.04) based on a significance level of p<0.01.

One previous meta-analysis [135] found that alcohol consumption was not significantly associated with lung cancer or prostate cancer worldwide. We similarly found no significant association between alcohol consumption and the risk of lung and prostate cancers in the Chinese population. However, another meta-analysis of international studies found that consumption of beer and hard liquor was a risk factor for the development of lung cancer in males (pooled ORs = 1.46 and 1.34) [20]. Our subgroup analysis did not identify alcohol consumption as a risk factor for Chinese males (p = 0.09). It appears that the effect of alcohol consumption on lung cancer may vary based on the type of alcohol consumed, the ethnicity of the study subjects, or on other lifestyle factors of the participants.

International meta-analyses on the effect of alcohol consumption on pancreatic cancer have provided inconsistent results. Bagnardi et al. found no significant association between alcohol and pancreatic cancer [136], but Tramacere et al. found that heavy drinking was a risk factor for pancreatic cancer (pooled RR: 1.22, 95% CI: 1.12–1.34) [140]. Another meta-analysis of Chinese studies also found that heavy drinking was a risk factor for pancreatic cancer in males (OR = 1.58, 95% CI: 1.01–2.34) [141]. Our meta-analysis found no significant (p = 0.04) association between alcohol consumption and pancreatic cancer based on a significance level of p<0.01. Our subgroup analysis similarly revealed that alcohol consumption was a significant risk factor for pancreatic cancer among Chinese males (pooled OR = 1.71, p<0.00001).

There are few studies on the association between alcohol drinking and breast cancer among Chinese people. Interestingly, our meta-analysis of four studies found that alcohol consumption is a protective factor for female breast cancer (pooled OR = 0.76) in the Chinese population. Previous meta-analyses of international studies on non-Chinese populations) reported a positive association between alcohol consumption and breast cancer [136], [142]–[143]. One study reported that the risk factors for breast cancer varied among different ethnic groups [144]. These findings suggest that alcohol may play different roles in the development of breast cancer in different ethnic groups or people with different lifestyle, environmental, behavioral and genetic factors. Further studies are needed to determine which of these differences are responsible for the differences in risk.

We also identified studies of the effect of alcohol consumption on the risk of developing cancer of the ampulla of Vater cancer, nasopharyngeal cancer, oral cancer, gallbladder cancer and ECC. Our meta-analyses found that alcohol was a significant risk factor for nasopharyngeal and oral cancers in Chinese people (pooled ORs = 1.21 and 1.71 respectively), that it was a protective factor for gallbladder cancer, and that no significant association existed between alcohol consumption and the other two cancers. However, these results need to be confirmed in further studies because the sample size and the number of studies on these cancers covered in this review were relatively small (see Table 1).

The interaction of alcohol consumption and other risk factors was not studied in sufficient detail, and the results are inconsistent in the present study. There is some evidence to suggest that alcohol and smoking have a greater relative (even synergistic) effect together than alone [6]. It seems that alcohol drinkers are more likely to be smokers, so it is valuable to clarify whether the two habits interact with each other with regard to the incidence of various cancers.

The strengths and limitations of this review include the following. First, bias may have been introduced because non-published data and papers published in languages other than English and Chinese were not included and because some studies without sufficient data to calculate the OR or RR were excluded. Second, the strengths of our review include the large number of subjects investigated and the comprehensive picture provided. However, the lack of uniformity among the definitions of drinking and the inconsistent classification of ethanol doses and the duration or frequency of alcohol consumption are serious weaknesses in the primary studies. Third, although we conducted a subgroup analysis, there was still heterogeneity between the studies, possibly due to the differences among the definitions of alcohol consumption. The sensitivity analysis indicated the results of our meta-analysis were relatively consistent even when some studies were excluded. Last, this present review primarily involved case-control studies published in the existing literature, and the results should be interpreted cautiously due to the recall bias of case-control studies. Therefore, further prospective studies based on intensive designs are needed.

Implications for future practice and study: A previous study found that there was a clear decrease in risk with longer periods of abstention from alcohol consumption [39]. Given that alcohol consumption, and even heavy drinking, is so common and is an inveterate habit in China, public health programs and national policy responses including legislation, education, and the organization of alcohol control activities are important for cancer prevention in the Chinese population. We would like to emphasize the importance of adopting a consistent definition of current, former, ever, and never drinking and the need to be explicit when presenting the data. Uniformity is also important if a study reports the drinking dose, duration, and frequency; uniformity facilitates making comparisons between studies of different populations and regions. Furthermore, future studies need to focus on the interaction of alcohol consumption with other common risk factors, such as tobacco use.

In conclusion, the effect of alcohol consumption on the risk of some cancers might vary with ethnicity, the type of alcohol consumed, the drinking dose, or the lifestyle of the participants. For the Chinese population, alcohol consumption increased the risk for EC, gastric cancer, HCC, colorectal cancer, pancreatic cancer and NPC.

Public health programs and national policy responses to limit alcohol intake are needed to reduce the incidence of cancer in China. Further intensive studies based on well-designed schemes must focus on the interaction between alcohol consumption and common lifestyles, as well as the possible interaction with other risk factors, such as tobacco use.
